# Structure of potassium channels

**DOI:** 10.1007/s00018-015-1948-5

**Published:** 2015-06-13

**Authors:** Qie Kuang, Pasi Purhonen, Hans Hebert

**Affiliations:** Department of Biosciences and Nutrition, Karolinska Institutet, Novum, 14183 Huddinge, Sweden; School of Technology and Health, KTH Royal Institute of Technology, Novum, 14183 Huddinge, Sweden

**Keywords:** Selectivity, Conductivity, Gating, Sensor domain, RCK

## Abstract

Potassium channels ubiquitously exist in nearly all kingdoms of life and perform diverse but important functions. Since the first atomic structure of a prokaryotic potassium channel (KcsA, a channel from *Streptomyces lividans*) was determined, tremendous progress has been made in understanding the mechanism of potassium channels and channels conducting other ions. In this review, we discuss the structure of various kinds of potassium channels, including the potassium channel with the pore-forming domain only (KcsA), voltage-gated, inwardly rectifying, tandem pore domain, and ligand-gated ones. The general properties shared by all potassium channels are introduced first, followed by specific features in each class. Our purpose is to help readers to grasp the basic concepts, to be familiar with the property of the different domains, and to understand the structure and function of the potassium channels better.

## Introduction

Potassium (K^+^) channels locate in cell membranes and control transportation of K^+^ ions efflux from and influx into cells. They play crucial roles in both excitable and non-excitable cells and can be found in virtually all species, except for some parasites [1].

K^+^ channels have transmembrane helices (TMs) spanning the lipid bilayer. Based on the structure and function, the channels are categorized into three major classes: the voltage-gated (Kv) (six TMs), inwardly rectifying (Kir) (two TMs), and tandem pore domain (K2P) (four TMs) channels [2]. Furthermore, the ligand-gated (Kligand) channels have either two or six TMs and are stimulated by various messengers. A K^+^ channel can, independent of which class it belongs to, be divided into two parts: the pore-forming domain and the regulatory domain. The pore-forming domain is responsible for transportation of K^+^ ions and its structure is similar in all types of K^+^ channels. The regulatory domain senses diverse stimuli and its structure differs among the classes.

In this review, the structure of K^+^ channels is discussed (Table 1). The channel containing only the pore-forming domain is introduced first, followed by Kv, Kir, K2P, and Kligand channels. Since the pore-forming domain is shared by all of the channels, only specific features in each class are discussed. The function of one example in each class is given in more detail to demonstrate the importance of K^+^ channels for living cells.

**Table 1:** Structures included in this paper

Channel type	Number of TMs	Name	Organism	PDB
K^+^ channel pore	2TM	KcsA	*Streptomyces lividans*	1K4C [11] 1K4D [11] 2ATK [21] 3F5W [45] 3F7V [45] 3OGC [22]
Kv	6TM	Kv1.2	*Rattus norvegicus*	2A79 [54] 3LUT [63]
		Kv1.2–Kv2.1 chimera	*Rattus norvegicus*	2R9R [53]
		KvAP	*Aeropyrum pernix*	1ORQ [31] 1ORS^a^ [31] 2KYH^a^ [70]
Voltage-gated sodium channel	6TM	NavAb	*Arcobacter butzleri*	3RVY [131] 3RVZ [131] 3RW0 [131]
		NavRh	*Rickettsiales sp.* HIMB114	4DXW [132] 4EKW [134]
Transient receptor potential channel	6TM	TRPV1	*Rattus norvegicus*	3J5P [133] 3J5Q [37] 3J5R [37]
Kir	2TM	KirBac1.1	*Burkholderia pseudomallei*	1P7B [86]
		KirBac3.1	*Magnetospirillum magnetotacticum*	2WLH [97] 2WLI [97] 2WLJ [97] 2WLK [97] 2WLL [97] 2WLM [97] 2WLN [97] 2WLO [97] 2X6A [97] 2X6B [97] 2X6C [97] 3ZRS [90] 4LP8 [92]
		Kir3.1-KirBac1.3 chimera	Kir3.1: *Mus musculus* KirBac1.3: *Burkholderia xenovorans*	2QKS [87]
		Kir2.1	*Mus musculus*	1U4F^b^ [95]
		Kir2.2	*Gallus gallus*	3JYC [88] 3SPI [93]
		Kir3.1	*Mus musculus*	1U4E^b^ [95]
		Kir3.2	*Mus musculus*	3SYA [89] 3SYC [89] 3SYO [89] 3SYP [89] 3SYQ [89] 4KFM [91]
K2P	4TM	K2P4.1	*Homo sapiens*	3UM7 [108] 4I9W [109] 4RUE [104] 4RUF [104] 4WFE [103] 4WFF [103]
		K2P10.1	*Homo sapiens*	4BW5 [106] 4XDJ [106] 4XDK [106] 4XDL [106]
		K2P1.1	*Homo sapiens*	3UKM [107]
Kligand	2TM	MthK	*Methanobacterium thermoautotrophicum*	1LNQ [110] 2FY8^c^ [112] 3LDC^d^ [24] 3RBZ [111]
	6TM	MlotiK1	*Mesorhizobium loti*	3BEH^d^ [62]
	6+1TM	BKca	*Homo sapiens*	3MT5^c^ [115] 3NAF^c^ [113]
		BKca	*Danio rerio*	3U6N^c^ [114]
	6TM	Kch	*Escherichia coli*	1ID1^b^ [120]
K^+^ transporter	Separately expressed soluble protein	KtrA	*Bacillus subtilis*	2HMS^c^ [116] 2HMT^c^ [116] 2HMU^c^ [116] 2HMV^c^ [116] 2HMW^c^ [116]
K^+^ uptake system	Separately expressed soluble protein	TrkA	*Vibrio parahaemolyticus*	4J9U^e^ [124] 4J9V [124]
		TrkA	*Vibrio vulnificus*	4G65 [124]
K^+^ efflux system	The soluble domain of KefC at the C-terminus	KefC-CTD	*Escherichia coli*	3EYW^f^ [125] 3L9W^f^ [126]

^a^the structure contains only the sensor part

^b^the structure contains only the cytoplasmic domain

^c^the structure contains only the gating ring part

^d^the structure contains only the transmembrane part

^e^the structure is with its transmembrane partner

^f^the structure is with KefF

In cells many K^+^ channels are regulated by their own auxiliary subunits, which profoundly affect the K^+^ channel physiological activities. Since these subunits are specific to each channel and the structural information of them is less well known, they are omitted in this review. Interested readers can refer to (Kv [3], Kir [4], K2P [5], and Kligand [3, 6]) for more detailed information.

## Pore of potassium channels

The basic organization of K^+^ channels is a tetramer with each monomer containing one pore-forming domain. Four pore-forming domains comprise a pore through which the ions move [7]. The general structure of the pore-forming domain can be described by the transmembrane part of KcsA, a two TMs K^+^ channel from *Streptomyces lividans* [8]. The active site of K^+^ channels is composed of four conserved signature sequences, TVGYG^75−79^ functioning as a selectivity filter (SF) to conduct K^+^ ions. K^+^ ions are conducted very efficiently, at near diffusion-limited rates (10^7^ ions channel^−1^s^−1^) [9]. Simultaneously, K^+^ channels are highly selective and at least 10,000 times more permeant for K^+^ than sodium (Na^+^) ions [8]. Besides the feature with regard to selectivity and conductivity, K^+^ channels are tightly regulated. Another interesting property is that many K^+^ channels can be inactivated, meaning that they enter stable nonconductive states shortly after opening. One kind of inactivated state is closely coupled to a conformational change of the SF.

### Selectivity and conductivity

**Fig. 1:**
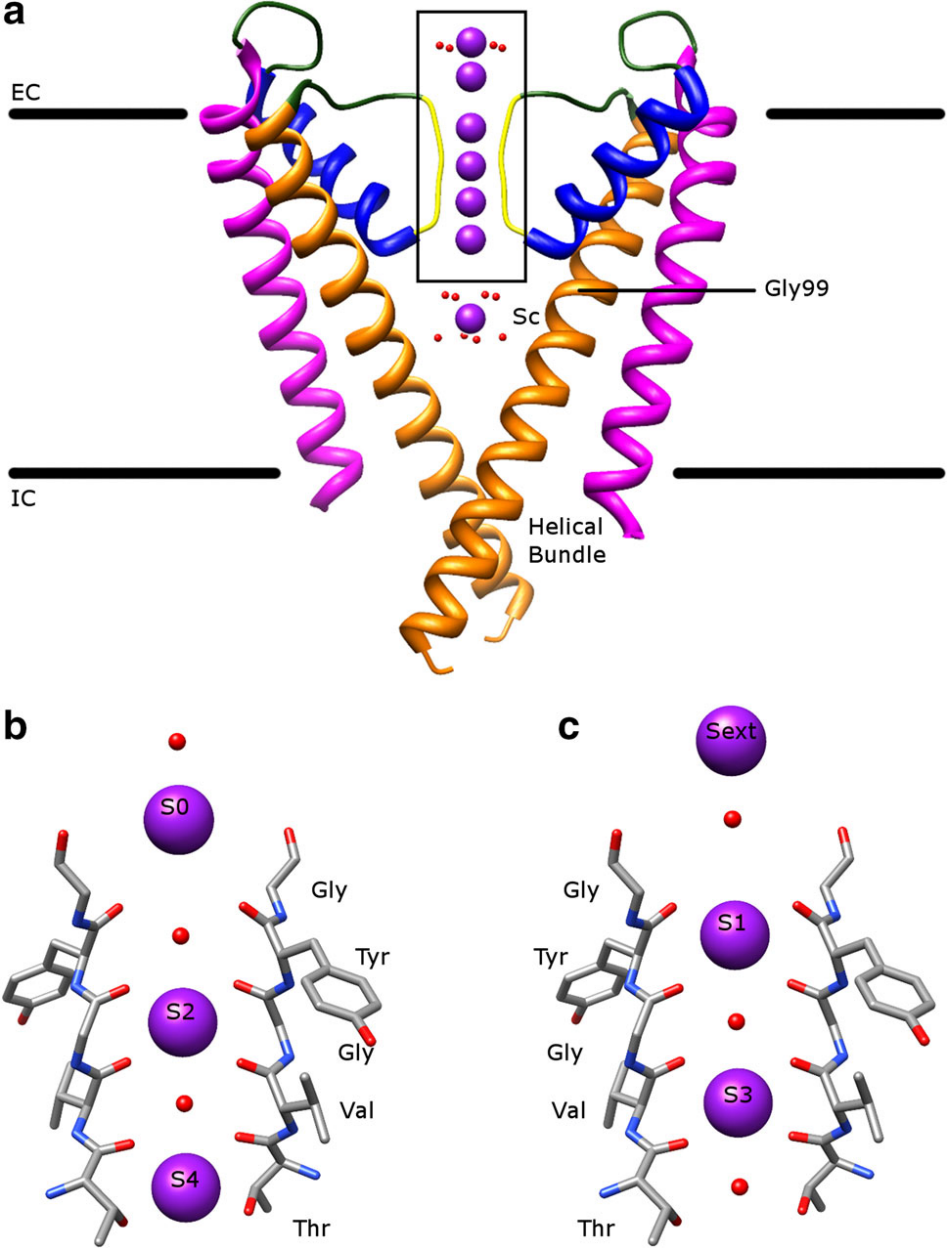
The transmembrane part of KcsA. **a** The atomic structure of KcsA in the conductive state (PDB: 1K4C) viewed along the membrane plane. The pore-forming domain consists of the outer helix (*magenta*), loop regions (*green*), pore helix (*blue*), SF (*yellow*), and inner helix (*orange*). The conducted K^+^ ions are represented by *purple balls* with surrounding water molecules in *red*. EC is extracellular and IC is intracellular for short. The glycine hinge (Gly99) and the helical bundle are labeled. **b**, **c** The enlarged view of the *boxed area* in (**a**) containing the SF and the extracellular entryway. The K^+^ ions are in two configurations, either in S2 and S4 (**b**) or S1 and S3 (**c**) during conduction. The water molecules occupy the vacant ion positions in S1 and S3 (**b**) or in S2 and S4 (**c**). Other ions are located in the extracellular entryway (either S0 (**b**) or Sext (**c**)) and in the central cavity (Sc (**a**)). For clarity, only two monomers opposite to each other are shown. The amino acid sequence of the SF is labeled. All figures (Figs. 1, 2, 3, 4, 5, 6, 7, 8) in this paper were made using Chimera [130] and GNU Image Manipulation Program (GIMP)

The mechanism of selectivity and conductivity of K^+^ channels is well studied in KcsA [8, 10, 11]. The structure of KcsA at high K^+^ ion concentrations (protein data bank (PDB): 1K4C [11]) is demonstrated in Fig. 1a. The K^+^ ions usually go from the intracellular side (helical bundle), then enter the central water-filled cavity (Sc), followed by passing the SF (S4–S1), and finally reach the extracellular entryway (S0 and Sext), down the electrochemical gradient. The K^+^ ions are hydrated in the central cavity, dehydrated in the SF, and then rehydrated in the extracellular entryway. The noticeable feature is that there are four evenly spaced K^+^ binding sites (S1–S4), which are formed by the carbonyl oxygens of TVGYG and the side chain of threonine. Four K^+^ ions can bind to these sites, where each K^+^ ion sits in the middle of two oxygen layers (Fig. 1b, c). Since the arrangement of these protein oxygens can mimic the water oxygens surrounding a K^+^ ion in solution, the transfer energy of the K^+^ ions from the central cavity to the SF is low [7]. The net result is that the conduction of K^+^ ions occurs at rates near the diffusion limit.

The proposal discussed above explains the mechanism of conduction perfectly and the selectivity could be explained by the fact that permeation of Na^+^ ions is energetically unfavorable, since the radius of a Na^+^ ion is smaller than a K^+^ ion [8, 12]. In fact, it has been reported that Na^+^ ions and their bound protein oxygens are in the same layer, allowing shorter ligand–ion distance [13]. A similar structure is observed for Li^+^ ions [14]. However, if K^+^ ions bind the S1–S4 sites tightly due to their high selectivity, how can they be released from these sites and transported across the membrane at a very fast speed? The near diffusion-limited rate of conduction suggests that K^+^ ions have weak binding in the protein. After analyzing the ion occupancy in the SF by different methods, MacKinnon and his colleagues proposed that only two ions exist in the SF at a time and they adopt two configurations, in which the ions are either in S2 and S4 (2,4-configuration, Fig. 1b) or in S1 and S3 (1,3-configuration, Fig. 1c) with two water molecules occupying the two corresponding vacant binding sites [10, 11, 15, 16]. The queue of ions and water molecules move in a concerted manner by either a concentration-independent path when the 2,4-configuration shifts to the 1,3-configuration or a concentration-dependent path when a third ion enters from one side of the SF and another ion exits from the opposite side [7, 10, 15]. The transfer energy cost between two configurations is expected to be low [10] and S2 favors binding of K^+^ ions, but not Na^+^ ions [17, 18]. The repulsion between two ions in the doubly occupied SF facilitates the conduction as well [7, 15].

K^+^ ions stabilize the conductive conformation of the SF, which in turn favors conduction of K^+^ ions [12, 19]. On the other hand, Na^+^ ions may stabilize a nonconductive state, which has a distorted structure of the SF (structure in low K^+^ ion concentrations, PDB: 1K4D [11]) [20]. The SF in the conductive state can conduct Na^+^ ions as well, when K^+^ ions are absent [20]. However, under physiological conditions, two K^+^ ions residing in the SF prevent Na^+^ ion conduction [20]. Thus, the SF and K^+^ ions work together to keep the selectivity and conductivity. Furthermore, the amino acid residues surrounding the SF could contribute to maintaining the conductive state of the SF [8, 12, 21–23]. In addition, other studies reveal that one or more of the following effects are involved to explain the selectivity and conductivity: different ion occupancies between two configurations [24], different binding sites favoring different ions [14, 17, 18, 25], ion concentrations in solution [26], the number of binding sites [27], the effect of the already bound ions in the SF [28], water molecules in the central cavity [29, 30], existence of the pore helix [8, 29], and different conformations of the SF [21, 22].

To summarize, the SF acts as an elegant machine to conduct K^+^ ions efficiently among other available ions. All factors that influence the integrity of the SF, even distant amino acid residues away from the SF, may change the selectivity and conductivity of the channel.

### Gating

K^+^ channels usually have three states: resting, activated, and inactivated. The channels are usually closed in the resting state, and opened after stimuli activation, followed by turning to the nonconductive states. The gating is the process to control closing and opening of the channel [31]. There are two kinds of gating mechanisms: the intracellular one is at a position where the inner helix bends and the extracellular one includes the SF [32]. These two gates are coupled, but the effects of coupling vary in different K^+^ channel classes. For example, the two gates in Kv channels (introduced in the “Voltage-gated potassium channels”) are negatively linked to make them easily enter the inactivated states, whereas the two gates in the K2P channels (introduced in the “Tandem pore domain potassium channels”) are positively coupled to facilitate their constitutive opening [33]. The coupling is involved in the arrangement of the K^+^ channel, mainly in the area at the lower part of the SF [32, 34–37].

#### The intracellular gate

**Fig. 2:**
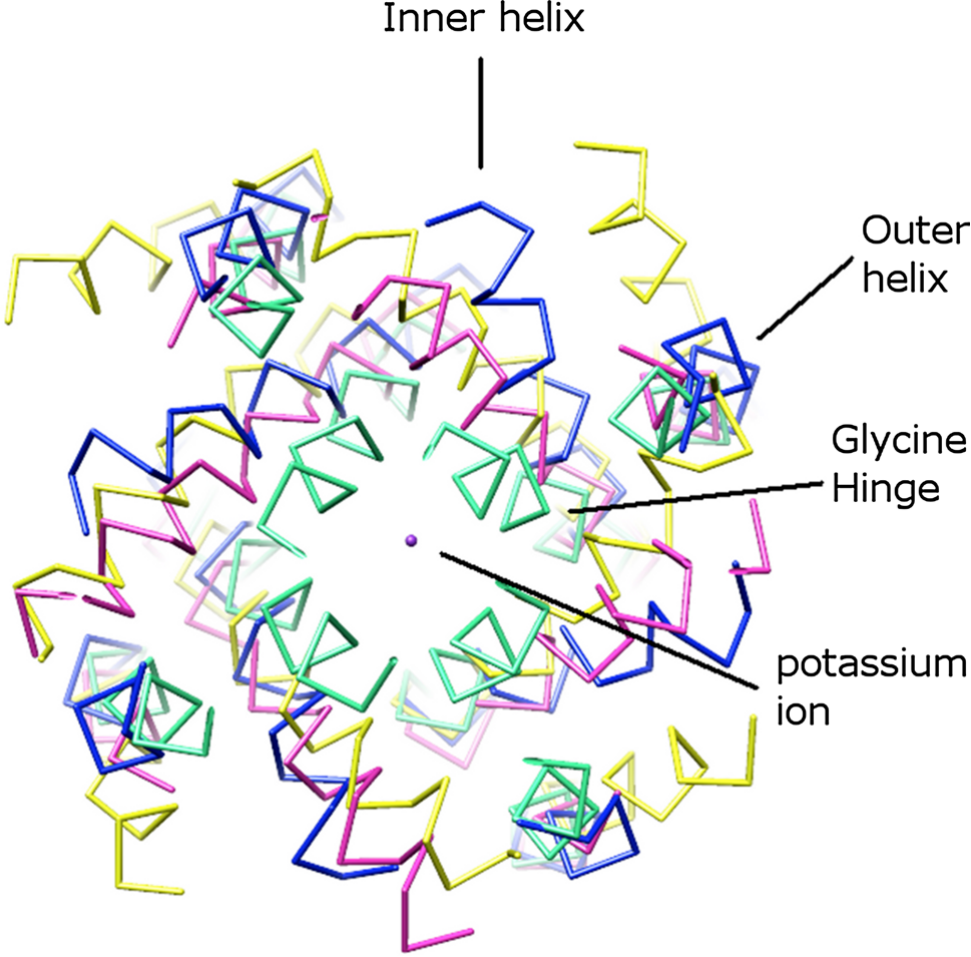
Structural comparison of bacterial K^+^ channels: KcsA (PDB: 1K4C, *cyan*), KcsA (PDB: 3F5W [45], *magenta*), KvAP (PDB: 1ORQ, *yellow*), and MthK (PDB: 3LDC [24], *blue*). Except for KcsA in the closed conformation (*cyan*), the others are in open conformation. All structures are viewed from the intracellular side. The glycine hinges are in a similar position in these proteins. The outer helix, inner helix, and a queue of K^+^ ions are labeled

The KcsA structure described above (PDB: 1K4C) is in a closed conformation, where the inner helices cross near the intracellular membrane interface (Fig. 1a). The open structure is represented by MthK, a calcium-gated K^+^ channel from *Methanobacterium thermoautotrophicum*, in which the inner helices are bent and splayed out following a glycine residue [38]. This glycine hinge is observed in other K^+^ channels, such as in KvAP, a voltage-gated K^+^ channel from *Aeropyrum pernix* [31]. Figure 2 shows the structural comparison of KcsA, KvAP, and MthK. The glycine hinges (G99 in KcsA, G220 in KvAP, and G83 in MthK) are located in a similar position in these proteins. In many eukaryotic Kv channels, this glycine hinge is replaced by P-X-P, where P is proline and X is any amino acid residue [39]. P-X-P allows the sixth helix (S6, corresponding to the inner helix in KcsA) to bend to interact with the linker between the pore-forming domain and the voltage sensor domain. In this way, the signal can be transferred from the voltage sensor domain to the pore-forming domain [40].

#### The extracellular gate

Since the SF controls conduction of K^+^ ions, it can serve as a gate at the extracellular side as well. Indeed, the SF adopts different structures in the resting, activated, and inactivated states [32]. Understanding the structure of inactivation is of considerable clinical and pharmaceutical interest. In Kv11.1, this process is critical for normal cardiac repolarization and disturbance is related to unintended side effects of arrhythmia and sudden death [41].

There are two mechanisms for inactivation of the K^+^ channels: N- and C-types. The former is a fast autoinhibitory process, existing in some Kv channels, where the N-terminal part interacts with the open K^+^ channel and occludes it [42, 43]. In the proposed N-type inactivation model [42], the first three amino acid residues at the N-terminus (inactivation ball) bind to the central cavity, the following eight hydrophobic amino acid residues extend from the cavity to the intracellular entryway, and the subsequent nine hydrophilic amino acid residues interact with the aqueous protein surfaces of the cytosolic domain (T1–S1 linker region). A mutagenesis study supports the view that electrostatic interactions between the inactivation ball and the T1–S1 linker region facilitate N-type inactivation [44]. C-type inactivation is usually a slow process and results from conformational changes of the SF, together with elimination of K^+^ ions and water molecules [32, 45]. The nonconductive structure obtained at low K^+^ ion concentrations (PDB: 1K4D) may represent an inactivated conformation [46]. In fact, the SF of this structure (PDB: 1K4D) resembles the one from an open-inactivated structure (PDB: 3F7V [45]), although the intracellular gate is closed in the former and open in the latter.

**Fig. 3:**
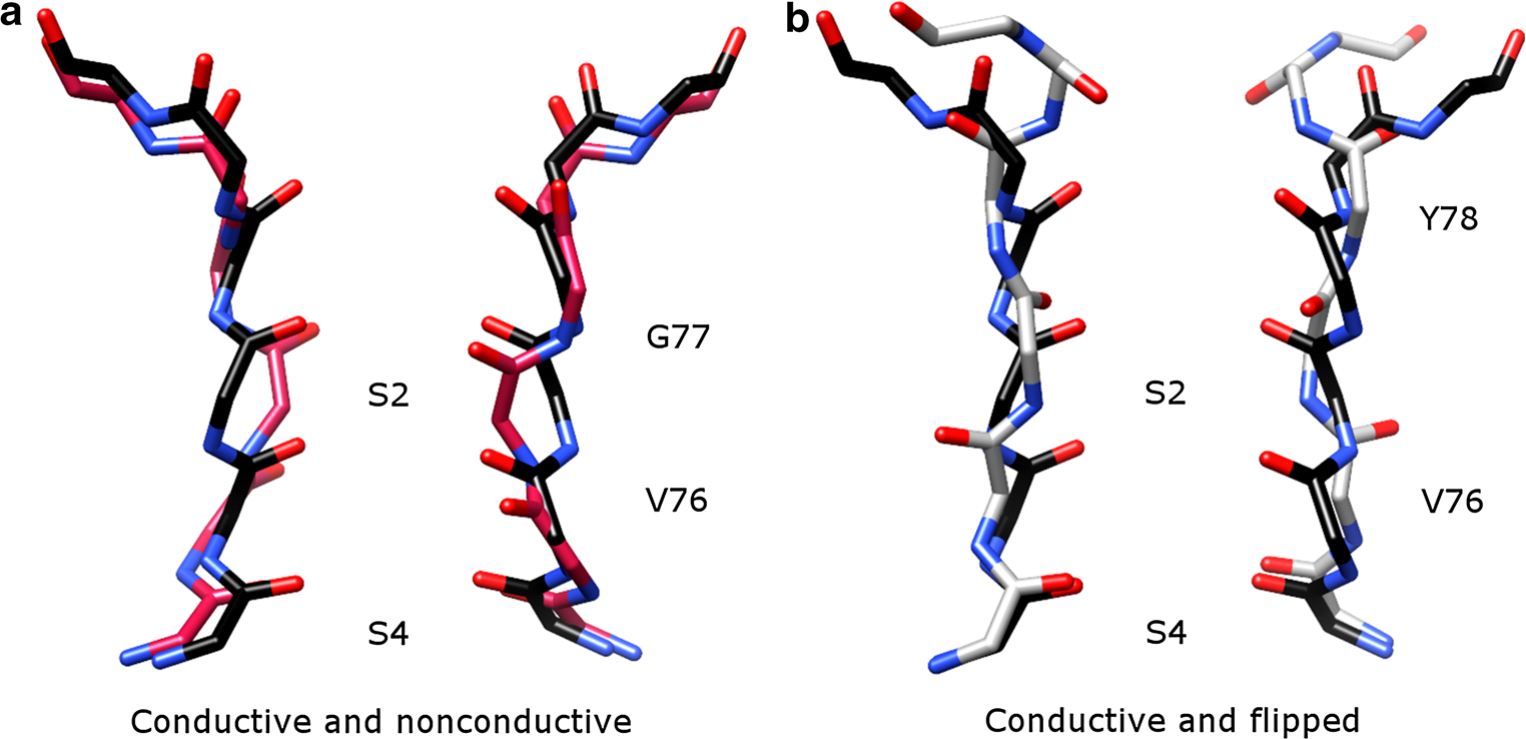
Activated, inactivated, and flipped SF structures of KcsA, viewed along the membrane plane. **a** Comparison of conductive (PDB: 1K4C, *black*) and nonconductive (PDB: 1K4D, 3F7V and 3F5W resemble each other and 3F7V is shown in *magenta*) structures. V76 and G77 are reorientated in the nonconductive state. **b** Comparison of conductive (PDB: 1K4C, *black*) and flipped (PDB: 2ATK [21] and 3OGC [22] are similar and 2ATK is shown in *gray*) structures. V76 and Y78 are reorientated in the flipped conformation. The S2 and S4 binding sites are labeled

The majority of K^+^ channels, including both prokaryotic and eukaryotic ones, undergo C-type inactivation [36]. A molecular mechanism is proposed based on a series of KcsA structures with different degree of opening of the intracellular and extracellular gates [45]. When both gates are opened, the channel conducts K^+^ ions. As the intracellular gate opens to a certain degree (the distance between two T112 residues on diagonally positioned inner helices >17 Å), the SF starts to change its conformation from the conductive (activated) state to the nonconductive (inactivated) state (Fig. 3a). The first step may be pinching of the G77 backbone carbonyls together. The rearrangement of oxygens destabilizes the ion in the S2 site, which might further destabilize the ion in the S3 site and narrow V76 as well. As a result, ions only occupy the S1 and S4 sites and two amino acid residues in the SF (G77 and V76) are reorientated. This prevents conduction of K^+^ ions in the nonconductive state. Another noticeable SF structure, in the so-called flipped conformation (Fig. 3b) is reported from the E71A mutant of KcsA [21, 22]. Whether this conformation represents a conductive [22] or a nonconductive state [21] has not received agreement yet. The presence of negatively charged lipids has been reported to be important for ion conduction in KcsA [47, 48] and binding of these lipids may decrease C-type inactivation [48].

## Voltage-gated potassium channels

The ability to learn, memorize, and perceive depends on the exchange of signals among neurons [49]. The action potential, the electrical signal generated by nerve cells involves several types of voltage-gated ion channels [50]. Our understanding of the action potential is based on the analysis of the squid axon [51], where voltage-gated Na^+^ (Nav) channels open for a short period followed by a rapid inactivation; after a short while, Kv channels are activated and remain open for a longer period [49, 50]. The net result is that when the action potential travels on depolarization, Na^+^ ions influx to the cell and K^+^ ions efflux to the extracellular environment. Action potentials have different functions in the neuron cell bodies and in the axons; in addition, various types of neurons have their own patterns of action potentials [50].

**Fig. 4:**
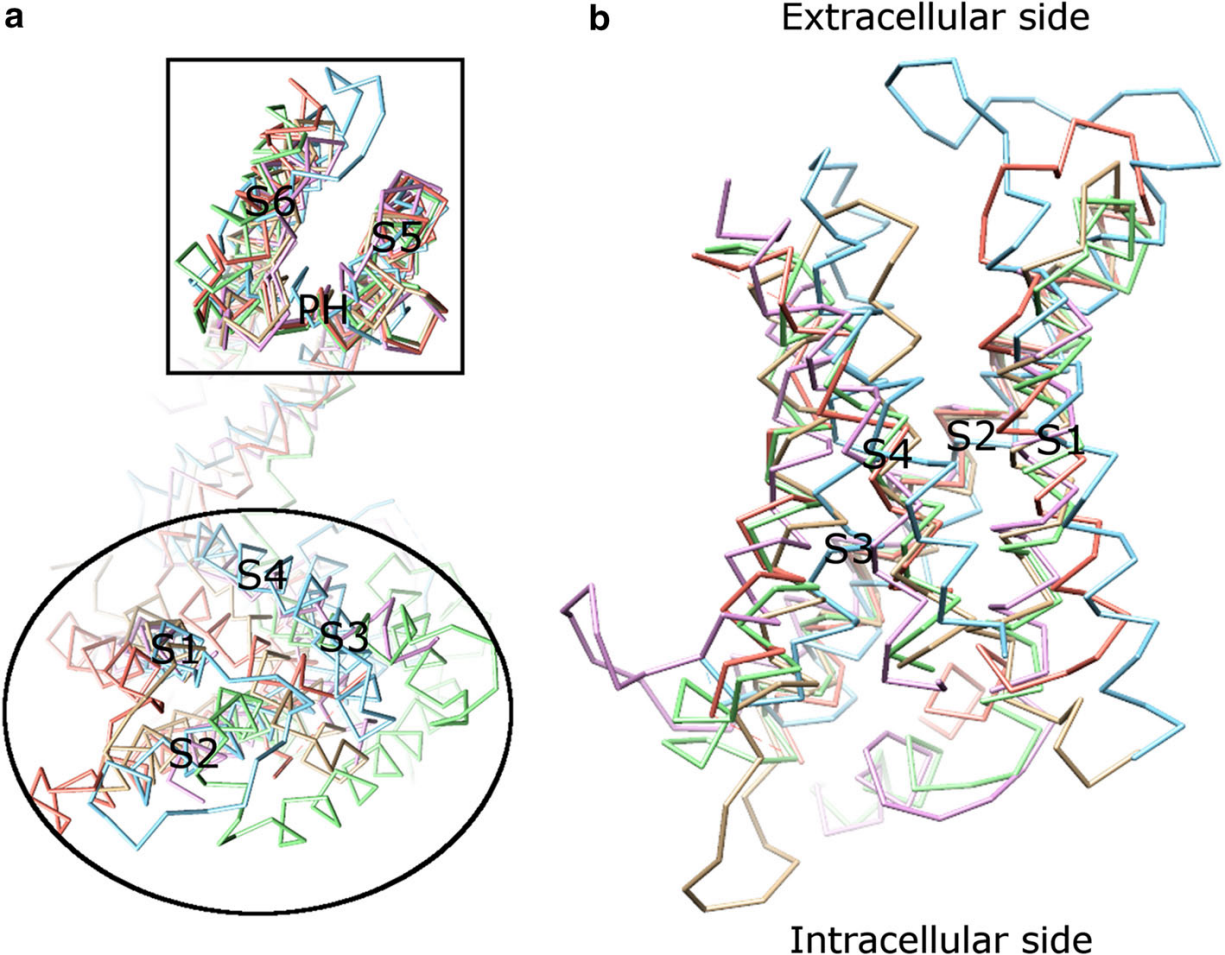
The VSDs in channels. **a** Alignment of monomers of different channels, viewed from the extracellular side. When pore-forming domains are aligned, the VSDs adopt various orientations. The VSD (in an *ellipse*) is composed of the first four helices (S1–S4). The pore-forming domain (in a *box*) consists of S5 (corresponding to the outer helix in KcsA in Fig. 1) and S6 (corresponding to the inner helix in KcsA in Fig. 1). The pore helix is labeled as PH. **b** Alignment of published VSDs structures, viewed along the membrane plane. **b** Shows an enlarged *side view*, rotated 90° from (**a**). Different VSDs are compared: Kv1.2 (a Kv channel from *Rattus norvegicus*, PDB: 3LUT, *light magenta*), MlotiK1 (a non-voltage-gated K^+^ channel from *Mesorhizobium loti*, PDB: 3BEH, *light brown*), NavAb (a Nav channel from *Arcobacter butzleri*, PDB: 3RVY [131], *light green*), NavRh (a Nav channel from *Rickettsiales sp.* HIMB114, PDB: 4DXW [132], *light orange*), and TRPV1 (a transient receptor potential channel from *Rattus norvegicus*, PDB: 3J5P [133], *light blue*). Although the VSDs adopt different orientations in the channels (**a**), they show a substantial overlap when only these domains are compared (**b**). The VSDs in PDB: 3LUT, 3RVY, and 4DWX are aligned best. Two loop regions between S1–S2 and S3–S4 in PDB: 3LUT are omitted. The resembling structures (PDB: 2A79, 2R9R, 3RVZ [131], 3RW0 [131], 4EKW [134], 3J5R [37], and 3J5Q [37]) are not depicted. The VSD of KvAP is not shown either, since the solved structure is either distorted (PDB: 1ORQ) or resembles (PDB: 1ORS [31] and 2KYH [70]) the one in Kv1.2 (PDB: 3LUT)

The majority of Kv channels open when the membrane is depolarized (less negative inside) and close when the membrane is hyperpolarized (more negative inside) [52]. The Kv channel has six TMs (Fig. 4a) and the first four helices (S1–S4) form the voltage sensor domain (VSD) [31, 40, 53, 54]. The last two helices (S5–S6, corresponding to the outer and inner helices in KcsA, respectively) form the pore-forming domain. The VSD senses the membrane potential alteration, followed by a conformational change that is coupled to gate the pore-forming domain.

### Voltage sensor domain

Considerable evidence supports the idea that the VSD functions as an independent domain. This domain is portable among K^+^ channels [55–57] and also exists in Nav and voltage-gated calcium (Cav) channels [58], the voltage-gated proton channel 1 (Hv1, also known as VSOP) [59, 60], and the voltage-sensor-containing phosphatase [61]. In addition, the solved structures (PDB: 2A79 [54], 2R9R [53], 3BEH [62], and 3LUT [63]) show that the VSDs are located at the periphery of the channel and are weakly attached to the pore-forming domain. Furthermore, although the structure of the VSD in KvAP (PDB: 1ORQ [31]) resembles the ones in other K^+^ channels (PDB: 2A79, 2R9R, and 3LUT), its position is distorted as compared to others [64]. The structural comparison of published VSDs including those in Nav and transient receptor potential channels shows that the VSDs adopt different orientations (Fig. 4a), although the structures themselves in these channels show a substantial overlap (Fig. 4b).

**Fig. 5:**
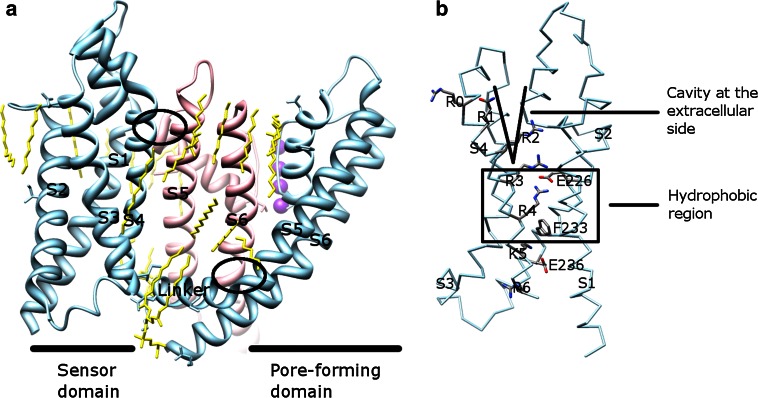
Kv1.2–Kv2.1 chimera channel (PDB: 2R9R) and its VSD. **a** The entire channel. The linker in one subunit (*light blue*) locates below the pore-forming domain of another subunit (*pink*). Two interfaces are in *ellipses*. The lipids (*yellow*) surround the channel and fill into the empty space between the pore-forming domain and the VSD. Each individual TM is labeled. **b** The VSD structure. The positive residues, the counterbalanced negative residues, the hydrophobic region (in a *box*), and the cavity at the extracellular side are labeled. **a** and **b** are viewed along the membrane plane

In the native conformation (PDB: 2A79, 2R9R, 3BEH, and 3LUT), the sensor domain from one subunit loosely contacts with the pore-forming domain from the adjacent subunit. These two domains interact in two coevolved interfaces (Fig. 5a) [65]. The first part is where the S4–S5 linker and S6 interact. The second part is where S1 in the VSD and the pore helix in the pore-forming domain interact. The first one is on the intracellular side and the second one is on the extracellular side. The large empty space between the VSD and the pore-forming domain is occupied by lipids (PDB: 2R9R), which play important functional and structural roles for Kv channels [52, 53, 64]. The VSD of Kv1.2 channels in the open conformation (PDB: 2A79, 2R9R, and 3LUT) contains a water-filled crevice at the extracellular side, followed by a hydrophobic region (Fig. 5b) [52, 53, 63].

### The positive charges and the paddle motif in the voltage sensor domain

Positively charged amino acid residues (arginine and lysine) in S4 make Kv channels electrically sensitive. In Shaker, a well-studied Kv channel from *Drosophila melanogaster*, approximately 13 positive charges are displaced across the membrane during activation [39, 66]. Up to eight positively charged residues have been found in each S4 and this number varies among different channels [1]. Shaker has seven such residues, denoted as R1-R4, K5, R6, and K7, of which R1-R4 contribute to most charge movement during activation [52, 66]. The positively charged residues are separated by hydrophobic residues and this triplet residue pattern (one positively charged residue and two hydrophobic residues) is evolutionarily conserved [1] and necessary for voltage sensing. The triple residue pattern, rather than the specific sequence, accounts for charge translocation [57]. The positive charges are counterbalanced by several negatively charged residues in other helices (E183, E226, E154, E236, and D259 in the Kv1.2–Kv2.1 chimera channel, based on PDB: 2R9R). The electrostatic interactions are believed to assist the movement of S4 in the membrane bilayer.

A conserved helix–turn–helix motif composed of the second helix in S3, S3b, and the N-terminal half of S4 is proposed to move together during activation [31, 67]. This motif, which contains R1–R4, was termed as the voltage sensor paddle. Similar to the entire VSD, the paddle motif is portable [53, 56, 57], suggesting that this motif resides in a relatively unconstrained environment. Although S3b and S4 may couple together as proposed in KvAP, some accessibility experiments suggest that S3 and S4 are movable relative to each other [68, 69]. One explanation for this contradiction could arise from the properties of VSD in different Kv channels. For instance, S3 breaks into S3a and S3b in KvAP (PDB: 1ORS [31] and 2KYH [70]), but is a continuous helix in Kv1.2 (PDB: 2A79, 2R9R, and 3LUT), whereas S3b is almost absent in KvLM (a Kv channel from *Listeria monocytogenes*) [71]. Other reasons are discussed in [39].

### Models for voltage sensing

VSDs exist in both depolarization- and hyperpolarization-activated Kv channels. It has been suggested that S4 has the same outward movement during activation, whereas the coupling between the VSD and pore-forming domain is opposite in these two kinds of channels [72, 73].

How does the VSD translocate the charges across the membrane when the membrane potential changes? The transporter, helical screw, and paddle motif models have been previously proposed to answer this question (reviewed in [39]). In all models, several explanations are shared: (1) translocation of the charges is carried out by the movement of the positively charged amino acid residues in S4; (2) S4 can rotate, translate, and adopt different helical conformations; and (3) the positive residues in S4 are counterbalanced by the negative residues in other helices in the VSD. The main differences are how far S4 has been moved vertically in the membrane and what environment S4 faces. With the atomic structures of Kv channels (PDB: 2A79, 2R9R, and 3LUT), as well as new biophysiological data (such as the observed omega current [74, 75] and proton leakage [76, 77]), a consensus model is appearing [39, 53, 63, 78]. In this focused electric field model (named in [63]), a hydrophobic region of approximately 10 Å thickness separates the external and internal solutions (Fig. 5b). Therefore, the charges only need to travel through the focused field, rather than across the entire membrane. Two structurally conserved negative residues in S2, which interact with a pair of positive residues in S4, are located at both sides of this hydrophobic region (Fig. 5b). E226 is on the extracellular side and E236 on the intracellular side (E226 and E236 are in the Kv1.2–Kv2.1 chimera channel, based on PDB: 2R9R).

In the resting state, R1 interacts with E226 and R4 with E236 [78]. On depolarization, S4 moves upwards to the extracellular side. A pair of positive residues (R1–R2, R2–R3, and R3–R4) pass through the hydrophobic region sequentially and each time interact with E226 (and maybe E236 as well) [78]. At least two positively charged residues are required to maintain the voltage-gated function [57]. The activated conformation is evident from the solved Kv1.2 structures (PDB: 2R9R and 3LUT), in which R1–R2 expose to the extracellular crevice and interact with the lipid; R3–R4 stay in the hydrophobic region and interact with E226; and K5–R6 expose to the intracellular crevice and interact with E236 (Fig. 5b) [53]. The intracellular crevice is deduced to be significant in the resting state, but may vanish in the activated state [53, 78]. The helical segment of S4 adopts a 3_10_-helical conformation when passing through the hydrophobic region [53]. This special helical conformation extends the helical length of S4 so that the paired positive residues can have the same orientation and interact with E226. The stability of this conformation may be affected by the protein environment from other helices in the VSD as well [79]. The S4 segment outside the hydrophobic region at both sides retains its α-helical conformation and the length of the 3_10_-helix may vary in different states [80]. F233 is proposed to have a structural role for facilitating formation of the electrostatic interactions between the positively and negatively charged residues [53, 81, 82]. The electrostatic interactions between the charged residues in Kv channels may be replaced by the hydrogen bonds in the non-voltage-gated K^+^ channels [83].

The structure of a Kv channel in the resting state has not been determined experimentally yet. The models are generated by computer simulation with the restriction from the available biochemical and biophysical data (e.g., [78, 80, 82]). Although these models generally agree with the current interpretation of the experimental data, the resting structure is still in question due to several uncertainties, such as the multiple sub-states of the channel and the network interactions between the charged residues. Thus, the focused electric field model, proposed interactions, and the computationally simulated structures may still be fine-tuned in the future.

Lipids are closely associated with both the VSDs and the pore-forming domains of Kv1.2–Kv2.1 chimera channel (Fig. 5a). The phospholipid head groups interact with the positive charges in S4 in the activated structure (PDB: 2R9R) [53]. The functional studies showed that lipid composition causes the VSDs to switch conformations in KvAP: phospholipids are required for it to reach the activated state, and non-phospholipids could stabilize its VSDs in the resting state [84]. Thus, gating of Kv channels is both lipid- and voltage-dependent [53, 84].

## Inwardly rectifying potassium channels

Kir channels have diverse physiological functions in the cell, depending on their type and location, and are modulated by various mediators, such as ions, phospholipids, and binding proteins [4]. Kir channels can be divided into seven subfamilies (Kir1.x–Kir7.x, where x is the number of each member) based on their mediators and the properties of ion conduction [4]. In vivo, they can be either homo- or hetero-tetramers [4].

Kir6.x channels are sensitive to nucleotides and are involved in glucose homeostasis. In pancreatic β-cells, Kir6.x channels and their partners (sulfonylurea receptor (SUR) subunits) work together to control insulin secretion [4]. Mutations in the *Kir6.x* or *SUR* gene result in a range of diseases and in fact, drugs targeted to SUR are routinely used to treat type 2 diabetes [85]. Kir6.x channels together with the SUR subunits have also been found in cardiac, smooth muscle, and brain nerve cells [4].

The unique feature of Kir channels is that they conduct K^+^ ions on hyperpolarization, rather than on depolarization as in other K^+^ channels. The inward rectification occurs because they are blocked by intracellular magnesium ions and polyamines on depolarization, whereas these blockers are released on hyperpolarization to allow K^+^ ions to influx into the cell [4]. The Kir channel contains a pore-forming domain and a cytosolic domain, where the pore-forming domain is responsible for ion conduction while the cytosolic domain regulates the gating of the channel.

### Misaligned pore helices

**Fig. 6:**
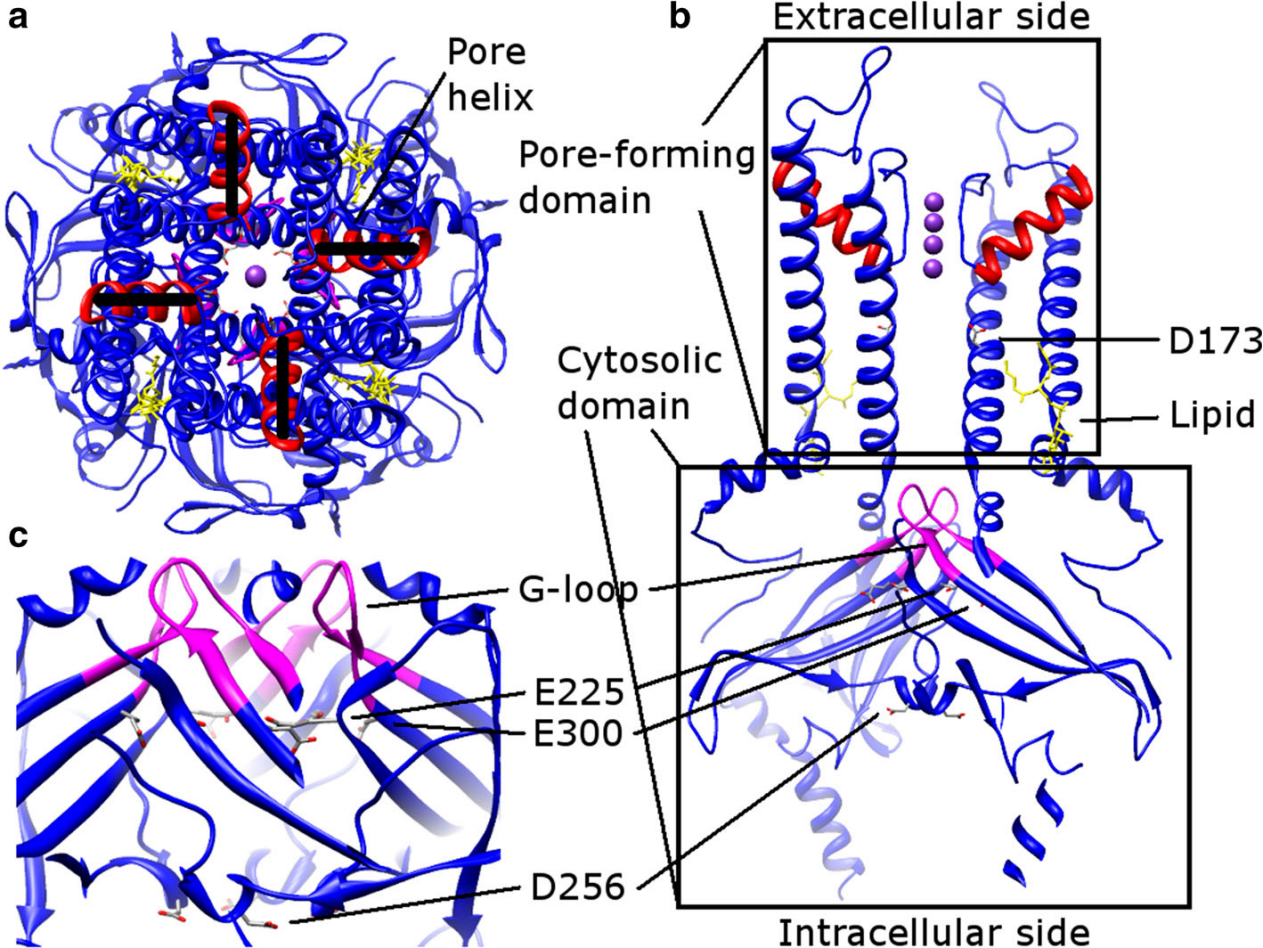
Kir2.2 structure (PDB: 3SPI). **a** Extracellular view. The pore helices (*red*) are misaligned. **b** Side view, rotated 90° from (**a**). The pore-forming domain locates above the cytosolic domain. The G-loop (residues from 301 to 311, *magenta*), and conserved multiple ion binding sites (*gray*) are labeled. The PIP_2_ lipids (*yellow*) are located at the interface between two domains. **c** The extended ion conduction pathway in the cytosolic domain, an enlarged view of (**b**)

In the first published Kir channel structure, the four pore helices in the pore-forming domain were misaligned (KirBac1.1, a Kir channel from *Burkholderia pseudomallei*, PDB: 1P7B [86]). Since this misalignment leads to destabilization of the central cavity, the authors suggested that KirBac1.1 structure represents a nonconductive state [86]. However, the same feature exists in all structures published later, including both prokaryotic and eukaryotic channels (Fig. 6a) (PDB: 2QKS [87], 3JYC [88], 3SYO [89], 3ZRS [90], 4KFM [91], and 4LP8 [92]). It is highly likely that the residues surrounding the pore helix and the SF determine this feature. One noticeable interaction is a salt bridge between E139 and R149 (the sequence is based on Kir2.2, a Kir channel from *Gallus gallus*, PDB: 3SPI [93] and 3JYC), which replaces the E71–D80 carboxyl–carboxylate interaction in KcsA (PDB: 1K4C). Thus, the network of interactions stabilizing the pore helix and the SF [21, 22] varies in different K^+^ channel classes [94].

### The cytosolic domain

The C-terminal cytosolic domain is rich in β-sheets and is located below the pore-forming domain, extending the ion conduction pathway. The cytosolic domain also forms a binding site to interact with diverse intracellular regulatory mediators [4]. Multiple ion binding sites (D173, E225, D256, and E300 in Kir2.2, Fig. 6b, c) in this domain are conserved and critical to inward rectification [86, 88, 95, 96]. In addition to the intracellular and extracellular gates in the transmembrane part, the Kir channel has a third gate (called G-loop), which is located at the apex of the cytosolic domain and forms a girdle around the central fourfold axis (Fig. 6b, c) [95]. This gate is intrinsically flexible and indeed, some solved Kir channel structures have these gates closed whereas others have them opened [86, 88–92, 95, 96].

Since the cytosolic domain is relatively independent from the pore-forming domain, it may adopt various conformations in the resting, activated, and inactivated states. An interesting paper illustrates a series of structures of KirBac3.1 (a Kir channel from *Magnetospirillum magnetotacticum*), which adopts twist, non-twist, latched, unlatched, and semi-latched conformations [97]. Reorientation and rotational movement of the cytosolic domain correlate with the ion configuration in the SF as well as binding of polyamine [97]. Growing evidence supports the view that rotation of this domain facilitates gating of the channel, although the rotational angles deviate in prokaryotic [90, 92] and eukaryotic [91] channels.

### Lipid regulation

Activation of the Kir channels depends on the signaling lipid phosphatidylinositol 4,5-bisphosphate (PIP_2_) [98]. As shown in Fig. 6b, PIP_2_ binds at the interface between the pore-forming domain and the cytosolic domain in Kir2.2. PIP_2_ induces a 6 Å translational movement of the cytosolic domain towards the membrane layer, concomitant with local conformational changes [93]. Interestingly, these changes are induced by binding of the head group of PIP_2_ to the cytosolic domain [93]. Besides Kir2.2, it has been observed that the cytosolic domain of Kir3.1 (a Kir channel from *Mus musculus*)-KirBac1.3 (a Kir channel from *Burkholderia xenovorans*) chimera in the open state gets closer to the pore-forming domain as compared to the closed state [87]. Although PIP_2_ usually activates eukaryotic Kir channels, it has an opposite effect on prokaryotic channels [99].

Kir3.2 (a Kir channel from *Mus musculus*, GIRK2) is activated by PIP_2_, as well as G proteins or Na^+^ ions [89]. Based on the structures from wild-type Kir3.2 with (PDB: 3SYA [89]) and without (PDB: 3SYO) PIP_2_, and R201A mutants with (PDB: 3SYQ [89]) or without (PDB: 3SYP [89]) PIP_2_, it was concluded that binding of PIP_2_ alone (in a similar position as in Kir2.2 shown in Fig. 6) does not open the gates in the pore-forming domain or the G-loop in the cytosolic domain, whereas G proteins alone open the G-loop but not the intracellular gate. When both PIP_2_ and G proteins are present, the channel opens [89]. Furthermore, it was proposed that the interaction between the Na^+^ ion and D228 could promote a similar conformational change as with G proteins [89, 100].

## Tandem pore domain potassium channels

K2P channels are abundant in both excitable and non-excitable cells, where they play diverse functions. The K2P channels are regulated by a variety of mediators, e.g., ions, pH, lipids, and regulatory proteins [2]. They set resting membrane potential and are targets of volatile anesthetics [5].

The TRAAK channel (TWIK-related arachidonic acid activated K^+^ channel, KCNK4, K2P4.1) exists exclusively in brain, spinal cord, and retina in mouse [101]. It is partially inhibited by barium ions at high concentrations, but is insensitive to other classical K^+^ channel inhibitors [101]. TRAAK can be stimulated by unsaturated fatty acids [5, 101]. Considering that these lipids exert both anti-ischemia and anti-convulsant effects, TRAAK channel was suggested to be a candidate in neuroprotection [5, 101, 102]. Indeed, riluzole, a neuroprotective drug is found to stimulate expression of the TRAAK channel [101]. Apart from the unsaturated lipids, the TRAAK channel can be activated by mechanical force, elevation of temperature, and alkalization from the intracellular side [5, 103, 104].

The K2P channels have several unique features. Functionally, the K2P channels are usually constitutively open [105], whereas the other K^+^ channels are tightly regulated in their closed and open states. Thus, the principle gating site might be the extracellular gate (see also C-type inactivation discussed in “The extracellular gate”) [104, 106]. Structurally, each mammalian K2P channel has four TMs and two pore-forming domains. Thus, the biological assembly of a K2P channel is a dimer [103, 104, 106–109].

### Human K2P structure

**Fig. 7:**
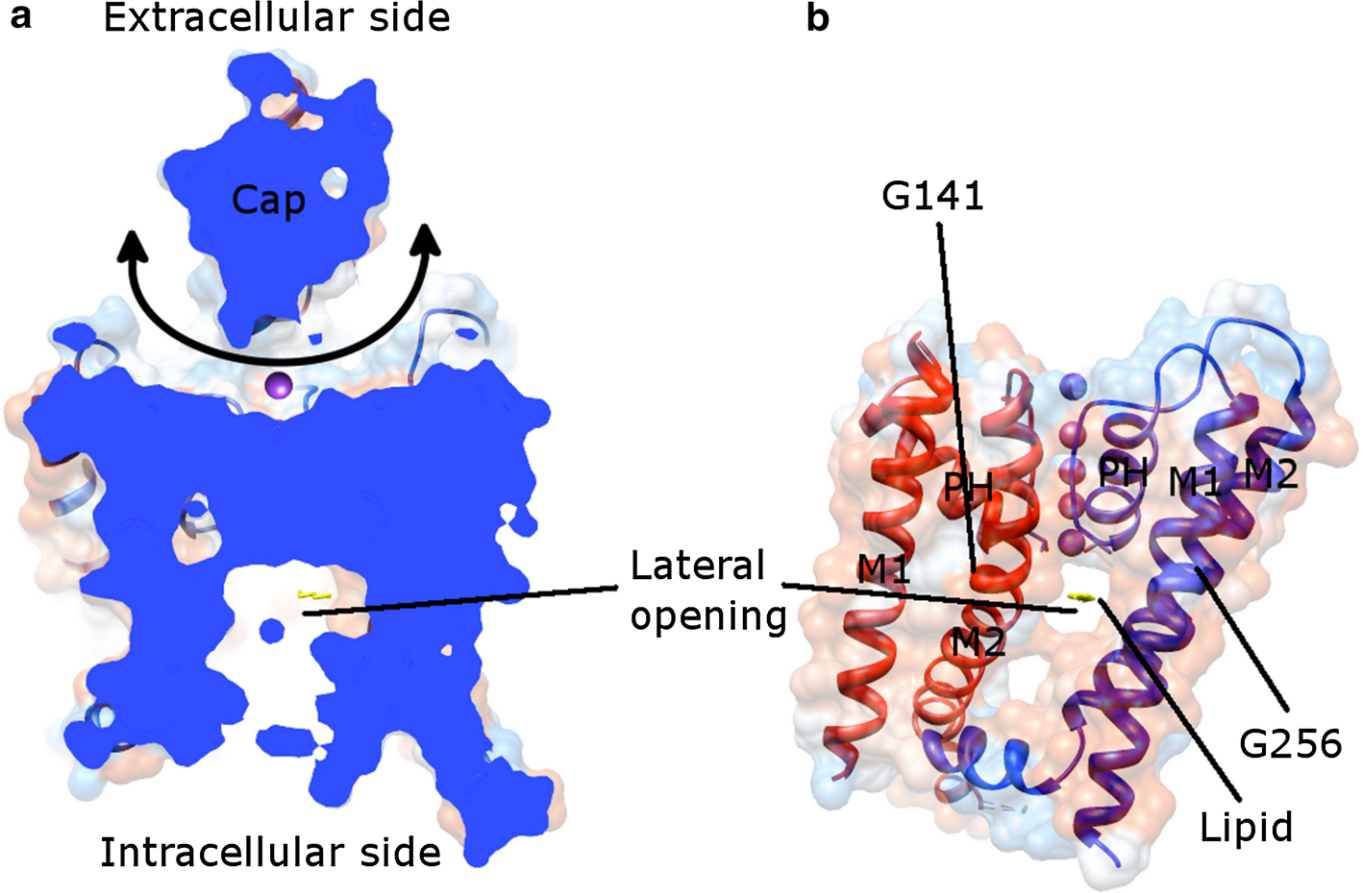
Human TWIK-1 structure (PDB: 3UKM) viewed along the membrane plane. **a** Cutaway view of the entire channel. The cap, a unique structure in K2P channels makes K^+^ ions (*purple*) coming laterally (indicated by a *double end* *arrow*). **b** Shows the lateral opening together with the channel model. Two adjacent subunits (*red* and *blue*) are shown. M1 (outer helix), M2 (inner helix), and PH (pore helix) are labeled, as well as the glycine hinges (G141 and G256). The structure presented here is in the down state. A lipid molecule (*yellow*) in the lateral opening is depicted. For clarity, the cap is omitted in (**b**) and only one lateral opening is displayed

The overall structure of human TRAAK (PDB: 3UM7 [108], 4I9 W [109], 4RUE [104], 4RUF [104], 4WFE [103], 4WFF [103]), TREK-2 (KCNK10, K2P10.1, PDB: 4BW5 [106], 4XDJ [106], 4XDK [106], 4XDL [106]), and TWIK-1 (KCNK1, K2P1.1, PDB: 3UKM [107]) resembles each other, regardless of the up and down movements of the inner helices in different states (see below). Each of them shows a rhomboid-shaped helical cap (Fig. 7a), which is not observed in other solved ion channel structures [108]. This cap is located on top of the transmembrane part of each K2P channel and is formed by the extracellular region within the first pore-forming domain, but is absent in the second pore-forming domain. In contrast to other K^+^ channels, K2P channels may exhibit domain swap where the units of the outer helix 1 (red M1 in Fig. 7b)-cap helix 1 (not shown in Fig. 7b) exchange between two subunits [103, 104, 109]. More importantly, the domain-swapped K2P channels are concluded to exist in the cell membranes [109]. The cap makes K^+^ ions accessible only from the bifurcated extracellular pathway [107, 108]. The coordination of ions in the SF in most structures resembles those from KcsA (PDB: 1K4C), except for those in the down state of TREK-2 (PDB: 4XDJ, 4XDK, and 4XDL) where only three ions were found in the SF.

### Up and down states of K2P

A series of K2P structures illustrates the gating/activation mechanism of the channel (PDB: 3UM7, 4I9W, 4RUE, 4RUF, 4WFE, 4WFF, 4BW5, 4XDJ, 4XDK, 4XDL, and 3UKM). The essence is the movement of the inner helix 2 (blue M2 in Fig. 7b). In the down state (PDB: 3UM7, 4WFF, 4XDJ, 4XDK, 4XDL, and 3UKM), the intracellular side of M2 is straight; whereas in the up state (PDB: 4I9W, 4RUE, 4RUF, 4WFE, and 4BW5), M2 is kinked approximately halfway through the membrane around the hinge glycine (G268 in TRAAK, G312 in TREK-2, and G256 in TWIK-1). The consequence of the up movement of M2 is that the lateral openings, which provide binding sites for regulatory lipids or for hydrophobic inhibitors to interact, are closed [103, 104, 106, 109]. Therefore, no lipid binds to the channel in the up state. On the other hand, lipids have been modeled in a similar position (Fig. 7b) in the down state of TRAAK (PDB: 4WFF), TREK-2 (PDB: 4XDJ), and TWIK-1 (PDB: 3UKM). The down state is believed to represent a nonconductive state, since the lipid occupies the central cavity thus blocking conduction of K^+^ ions [103, 106]. In accordance with the elevation movement of M2, other helices change their conformations [103, 104, 106]. These movements work together to gate the channel.

## Ligand-gated potassium channels

Most Kligand channels have either two or six TMs, and a cytosolic domain usually at the C-terminus, although some of them have additional TMs such as in BKca (a large conductance K^+^ channel from *Homo sapiens*, both voltage and calcium gated). The transmembrane part of a two TMs Kligand channel is similar to KcsA, and that of a six TMs resembles a Kv channel. The cytosolic domain in the Kligand channel acts as a receptor domain for binding of various messengers, including cAMP [62], calcium [110–115], and NADP [116, 117].

BKca participates in many biological processes including generation of action potentials, modulation of the tone of blood vessels, and release of hormones and neurotransmitters [118, 119]. It has been suggested that BKca can coassemble with multiple types of Cav channels to form macromolecular complexes in the central nervous system [50, 119]. Thus, complex formation with distinct Cav channels can tune the BKca activity and further control the diverse processes in which BKca is involved [119].

Although ligand-binding domains in diverse Kligand channels have their own structures and properties for interacting with various messengers, the general gating mechanisms among the channels are similar. The ligand-binding domains in BKca and its homologs are discussed in the following text as an example for other Kligand channels.

### The fourfold symmetric RCK octameric gating rings

BKca and MthK have large cytosolic domains, called RCK (the regulator of the conductance of K^+^ ion) [120]. The RCK domains modulate the function of some prokaryotic [110–112] and eukaryotic [113–115] K^+^ channels. Its homolog, KTN (K^+^ ion transport and nucleotide binding) domain also exists in prokaryotes for uptake of K^+^ ions [116, 117]. A prokaryotic genome analysis shows that a large number of two and six TMs K^+^ channels carry an RCK or a KTN domain, which illustrates a general theme utilized by this group of channels [1].

**Fig. 8:**
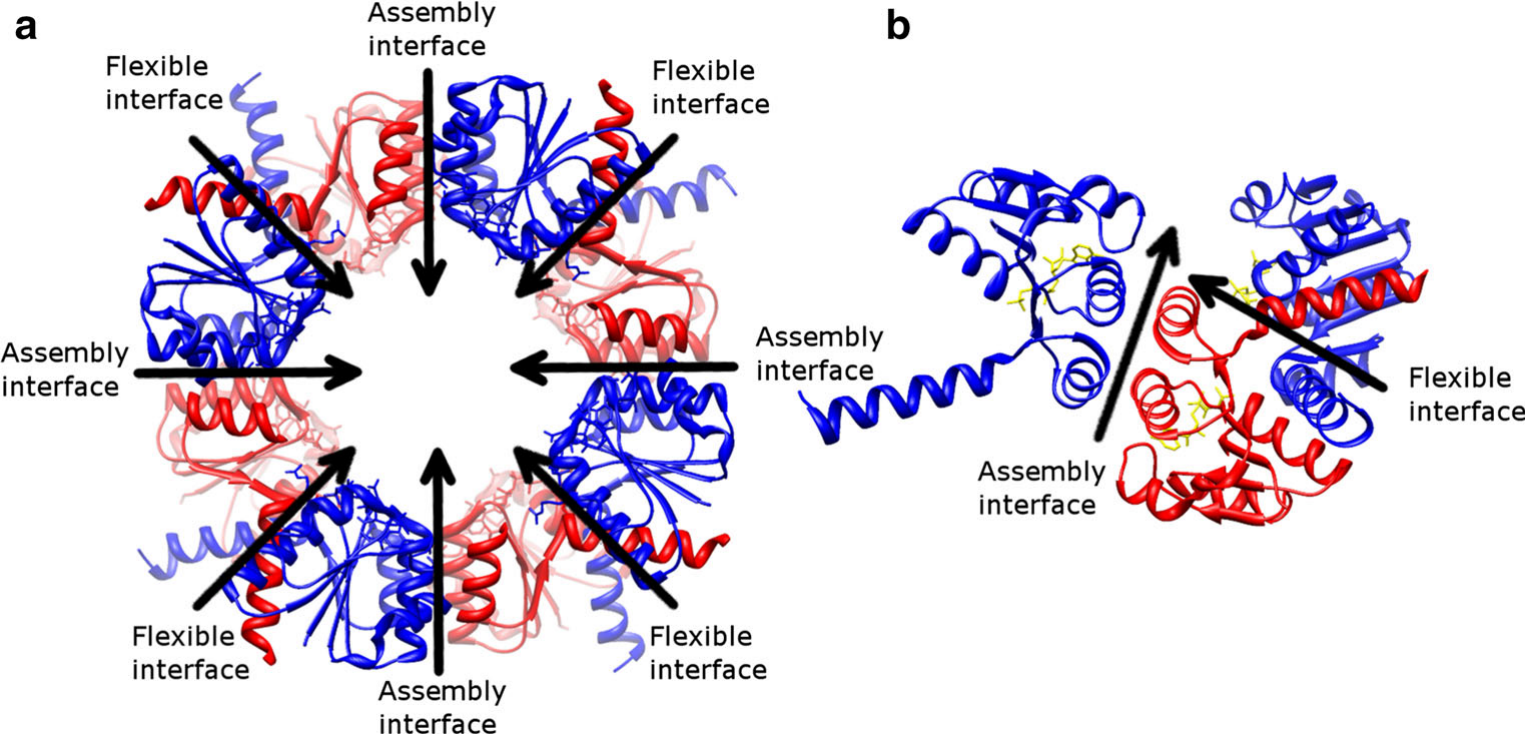
RCK gating ring of KtrA (PDB: 2HMW [116]). **a** Is viewed down the fourfold axis from the extracellular side. The RCK monomers in the top layer (*blue*) and bottom layer (*red*) are connected through the alternative flexible and assembly interfaces, but the monomers in the same layer do not interact with each other. (**b**) Is the side view, rotated 90° from (**a**). One RCK monomer (*red*) interacts with two adjacent monomers in another layer (*blue*). The adenosine triphosphate ligands are shown in *yellow*

The RCK domain can be expressed as an entire soluble protein through an internal methionine in the gene which encodes the full-length channel as well [110, 120–123]. Two soluble RCK proteins or one soluble RCK protein and one RCK domain from the full-length protein form an RCK dimer due to the strong interactions at the dimer interface (corresponding to the flexible interface in Fig. 8, this RCK dimer is also called a hinge dimer [117]) [120]. Four such dimers in BKca [113–115], MthK [110–112], and KtrA (a cytosolic subunit of a K^+^ transporter from *Bacillus subtilis*, with nucleotides as its ligands) [116] build up the octameric gating ring in an alternative arrangement (Fig. 8). The effect of ligand binding has been well documented in BKca and MthK, where different gating ring states have been determined [110–115]. In both proteins, the outer rim of the gating ring expands after binding of ligands. The free energy of ligand binding is transferred through a presumed rigid helical linker to the pore-forming domain and results in a conformational change of the SF to conduct K^+^ ions [112]. In BKca, only the layer facing the membrane undergoes a substantial conformational change and the other layer remains static [114]. On the other hand, both layers alter their arrangements in MthK after ligand binding.

### A dimer of dimer assembly of RCK

Growing references suggest that the RCK assembly is flexible and the fourfold symmetry observed in the octameric gating ring of BKca, MthK, and KtrA may not be valid in other RCK-containing proteins.

#### TrkA, a dimer of dimer assembly in two layers

The *TrkA* gene contains two tandem RCK domains. Four soluble TrkA proteins are assembled with their membrane proteins to form a main K^+^ uptake system in bacteria. In contrast to BKca that also contains two tandem RCK domains within one single gene, the recently reported TrkA gating ring adopts a dimer of dimer assembly (PDB: 4J9U [124]). In BKca, four RCK domains within the same layer of the octameric gating ring are identical, but different between two layers (See also the KtrA gating ring shown in Fig. 8. The blue and red RCK monomers are identical in KtrA, but homologous to each other in BKca). However, homologous RCK monomers exist in the same layer in TrkA, rather than in two layers as in BKca. Furthermore, as compared to the twisted structure from the isolated TrkA gating rings (PDB: 4J9V [124] and a comparably similar TrkA structure from another species, PDB: 4G65), the authors proposed that four assembly interfaces are not identical [124]. Two of them are static (fixed), and another two are flexible (mobile). Thus, different proteins may have their own properties of the assembly interfaces.

#### KefC-CTD, a dimer of dimer assembly in one layer

KefC is a K^+^ efflux system from *Escherichia coli* and bears a C-terminal KTN domain (KefC-CTD). Although the KTN domains have been observed to arrange into an octameric gating ring structure in KtrA [116], only one-layer assembly composed of four domains is proposed to be formed in KefC-CTD (PDB: 3EYW [125] and PDB: 3L9W [126]). Furthermore, instead of a fourfold symmetric layer as in BKca [113–115], MthK [110–112], and KtrA [116], two KefC-CTD hinge dimers are assembled together to form a dimer of dimer complex. As compared to the octameric gating ring structure, the assembly interfaces are disturbed due to existence of the helices 7 and 8 in KefC-CTD. This helix–turn–helix occupies the space in the assembly interface thus blocking formation of a higher assembly [125]. More interestingly, after binding of various ligands helices 7 and 8 are repositioned, which may alter the accessibility of the assembly interfaces [126]. The authors proposed that this one-layer assembly exists in the cell and the unidentified regulators or auxiliary proteins prevent formation of the octameric gating ring [125].

### Summary of the RCK assembly

The results of the RCK assembly have not received an agreement yet. Some show two-layer arrangements and some have one-layer assemblies. Several factors may explain the discrepancy. First of all, it is due to the structure of the RCK domains. Although all of the RCK domains share a similar Rossmann fold [120], each domain varies at its C-terminus, which affects the assembly as discussed in KefC-CTD. In addition, some RCK domains also have extra sub-domains, such as the C-terminal sub-domains in BKca, MthK, and TrkA. The effect of these extra domains remains to be elucidated [127]. Ligand binding changes the angle of the hinge dimer [112], as well as the order of the assembly [126]. The regulators or auxiliary proteins in the cell may further stabilize or prevent a certain assembly. Another important factor to be considered is the lipid environment for membrane proteins. Recent electron crystallographic studies of Kch (a six TMs K^+^ channel from *Escherichia coli*) suggested that the RCK domains expose to the solvent and do not interact with each other as observed in other octameric gating rings [128, 129]. Since the majority of studies concern only the soluble RCK domains, the resulting structures may be due to the artifacts where the restriction of the transmembrane partner is lacking. Besides the effect of absence of the transmembrane partner, the observed conformations may be affected by the crystal contacts as well [124]. Furthermore, the angle of the hinge dimer and its higher assembly may vary in different states (resting, activated, and inactivated) [125].

To summarize, both the hinge dimer and the higher arrangement between the dimers are flexible. The published results may be influenced by the experimental conditions. Other unidentified partners in the cell may affect the assembly as well.

## Perspective

Diverse biochemical and biophysical methods have been applied to understand the structure and function of potassium channels, besides X-ray crystallography that is still used as the main method to determine protein structures. Although considerable progress has been made, more studies are needed to explain the discrepancy in different reports, answer unclear questions, and aid in drug design. One important topic, the effect of the lipid environment may be worth investigating. Lipids not only provide a suitable environment for channels to fold, but also can participate in their activation. Another interesting topic is to study protein complexes, since membrane proteins often interact with other proteins to perform specific functions in the cell (such as the BKca/Cav complex mentioned above). In the future, more information and deeper understanding of channels will be obtained with developing techniques.

## References

[1] Kuo MM, Haynes WJ, Loukin SH, Kung C, Saimi Y (2005). Prokaryotic K(+) channels: from crystal structures to diversity. FEMS Microbiol Rev.

[2] Buckingham SD, Kidd JF, Law RJ, Franks CJ, Sattelle DB (2005). Structure and function of two-pore-domain K^+^ channels: contributions from genetic model organisms. Trends Pharmacol Sci.

[3] Pongs O, Schwarz JR (2010). Ancillary subunits associated with voltage-dependent K^+^ channels. Physiol Rev.

[4] Hibino H, Inanobe A, Furutani K, Murakami S, Findlay I, Kurachi Y (2010). Inwardly rectifying potassium channels: their structure, function, and physiological roles. Physiol Rev.

[5] Enyedi P, Czirjak G (2010). Molecular background of leak K^+^ currents: two-pore domain potassium channels. Physiol Rev.

[6] Zhang J, Yan J (2014). Regulation of BK channels by auxiliary gamma subunits. Front Physiol.

[7] MacKinnon R (2003). Potassium channels. FEBS Lett.

[8] Doyle DA, Morais Cabral J, Pfuetzner RA, Kuo A, Gulbis JM, Cohen SL, Chait BT, MacKinnon R (1998). The structure of the potassium channel: molecular basis of K^+^ conduction and selectivity. Science.

[9] Sansom MS, Shrivastava IH, Bright JN, Tate J, Capener CE, Biggin PC (2002). Potassium channels: structures, models, simulations. Biochim Biophys Acta.

[10] Morais-Cabral JH, Zhou Y, MacKinnon R (2001). Energetic optimization of ion conduction rate by the K^+^ selectivity filter. Nature.

[11] Zhou Y, Morais-Cabral JH, Kaufman A, MacKinnon R (2001). Chemistry of ion coordination and hydration revealed by a K^+^ channel-Fab complex at 2.0 A resolution. Nature.

[12] Lockless SW, Zhou M, MacKinnon R (2007). Structural and thermodynamic properties of selective ion binding in a K^+^ channel. PLoS Biol.

[13] Alam A, Jiang Y (2011). Structural studies of ion selectivity in tetrameric cation channels. J Gen Physiol.

[14] Thompson AN, Kim I, Panosian TD, Iverson TM, Allen TW, Nimigean CM (2009). Mechanism of potassium-channel selectivity revealed by Na(+) and Li(+) binding sites within the KcsA pore. Nat Struct Mol Biol.

[15] Zhou Y, MacKinnon R (2003). The occupancy of ions in the K^+^ selectivity filter: charge balance and coupling of ion binding to a protein conformational change underlie high conduction rates. J Mol Biol.

[16] Zhou M, MacKinnon R (2004). A mutant KcsA K(+) channel with altered conduction properties and selectivity filter ion distribution. J Mol Biol.

[17] Furini S, Domene C (2011). Selectivity and permeation of alkali metal ions in K^+^ -channels. J Mol Biol.

[18] Jensen MO, Borhani DW, Lindorff-Larsen K, Maragakis P, Jogini V, Eastwood MP, Dror RO, Shaw DE (2010). Principles of conduction and hydrophobic gating in K^+^ channels. Proc Natl Acad Sci USA.

[19] Krishnan MN, Bingham JP, Lee SH, Trombley P, Moczydlowski E (2005). Functional role and affinity of inorganic cations in stabilizing the tetrameric structure of the KcsA K^+^ channel. J Gen Physiol.

[20] Valiyaveetil FI, Leonetti M, Muir TW, Mackinnon R (2006). Ion selectivity in a semisynthetic K^+^ channel locked in the conductive conformation. Science.

[21] Cordero-Morales JF, Cuello LG, Zhao Y, Jogini V, Cortes DM, Roux B, Perozo E (2006). Molecular determinants of gating at the potassium-channel selectivity filter. Nat Struct Mol Biol.

[22] Cheng WW, McCoy JG, Thompson AN, Nichols CG, Nimigean CM (2011). Mechanism for selectivity-inactivation coupling in KcsA potassium channels. Proc Natl Acad Sci USA.

[23] Domene C, Furini S (2012). Molecular dynamics simulations of the TrkH membrane protein. Biochemistry.

[24] Ye S, Li Y, Jiang Y (2010). Novel insights into K^+^ selectivity from high-resolution structures of an open K^+^ channel pore. Nat Struct Mol Biol.

[25] Furini S, Domene C (2009). Atypical mechanism of conduction in potassium channels. Proc Natl Acad Sci USA.

[26] Renart ML, Triano I, Poveda JA, Encinar JA, Fernandez AM, Ferrer-Montiel AV, Gomez J, Gonzalez Ros JM (2010). Ion binding to KcsA: implications in ion selectivity and channel gating. Biochemistry.

[27] Derebe MG, Sauer DB, Zeng W, Alam A, Shi N, Jiang Y (2011). Tuning the ion selectivity of tetrameric cation channels by changing the number of ion binding sites. Proc Natl Acad Sci USA.

[28] Domene C, Vemparala S, Furini S, Sharp K, Klein ML (2008). The role of conformation in ion permeation in a K^+^ channel. J Am Chem Soc.

[29] Roux B, MacKinnon R (1999). The cavity and pore helices in the KcsA K^+^ channel: electrostatic stabilization of monovalent cations. Science.

[30] Furini S, Zerbetto F, Cavalcanti S (2007). Role of the intracellular cavity in potassium channel conductivity. J Phys Chem B.

[31] Jiang Y, Lee A, Chen J, Ruta V, Cadene M, Chait BT, MacKinnon R (2003). X-ray structure of a voltage-dependent K^+^ channel. Nature.

[32] Imai S, Osawa M, Takeuchi K, Shimada I (2010). Structural basis underlying the dual gate properties of KcsA. Proc Natl Acad Sci USA.

[33] Ben-Abu Y, Zhou Y, Zilberberg N, Yifrach O (2009). Inverse coupling in leak and voltage-activated K^+^ channel gates underlies distinct roles in electrical signaling. Nat Struct Mol Biol.

[34] Cuello LG, Jogini V, Cortes DM, Pan AC, Gagnon DG, Dalmas O, Cordero-Morales JF, Chakrapani S, Roux B, Perozo E (2010). Structural basis for the coupling between activation and inactivation gates in K(+) channels. Nature.

[35] Ader C, Schneider R, Hornig S, Velisetty P, Vardanyan V, Giller K, Ohmert I, Becker S, Pongs O, Baldus M (2009). Coupling of activation and inactivation gate in a K^+^ -channel: potassium and ligand sensitivity. EMBO J.

[36] McCoy JG, Nimigean CM (2012). Structural correlates of selectivity and inactivation in potassium channels. Biochim Biophys Acta.

[37] Cao E, Liao M, Cheng Y, Julius D (2013). TRPV1 structures in distinct conformations reveal activation mechanisms. Nature.

[38] Jiang Y, Lee A, Chen J, Cadene M, Chait BT, MacKinnon R (2002). The open pore conformation of potassium channels. Nature.

[39] Tombola F, Pathak MM, Isacoff EY (2006). How does voltage open an ion channel?. Annu Rev Cell Dev Biol.

[40] Long SB, Campbell EB, Mackinnon R (2005). Voltage sensor of Kv1.2: structural basis of electromechanical coupling. Science.

[41] Wang DT, Hill AP, Mann SA, Tan PS, Vandenberg JI (2011). Mapping the sequence of conformational changes underlying selectivity filter gating in the K(v)11.1 potassium channel. Nat Struct Mol Biol.

[42] Zhou M, Morais-Cabral JH, Mann S, MacKinnon R (2001). Potassium channel receptor site for the inactivation gate and quaternary amine inhibitors. Nature.

[43] Hoshi T, Zagotta WN, Aldrich RW (1990). Biophysical and molecular mechanisms of Shaker potassium channel inactivation. Science.

[44] Fan Z, Ji X, Fu M, Zhang W, Zhang D, Xiao Z (2012). Electrostatic interaction between inactivation ball and T1–S1 linker region of Kv1.4 channel. Biochim Biophys Acta.

[45] Cuello LG, Jogini V, Cortes DM, Perozo E (2010). Structural mechanism of C-type inactivation in K(+) channels. Nature.

[46] Yellen G (2002). The voltage-gated potassium channels and their relatives. Nature.

[47] Valiyaveetil FI, Zhou Y, MacKinnon R (2002). Lipids in the structure, folding, and function of the KcsA K^+^ channel. Biochemistry.

[48] Marius P, Zagnoni M, Sandison ME, East JM, Morgan H, Lee AG (2008). Binding of anionic lipids to at least three nonannular sites on the potassium channel KcsA is required for channel opening. Biophys J.

[49] Armstrong CM, Hille B (1998). Voltage-gated ion channels and electrical excitability. Neuron.

[50] Bean BP (2007). The action potential in mammalian central neurons. Nat Rev Neurosci.

[51] Hodgkin AL, Huxley AF (1952). A quantitative description of membrane current and its application to conduction and excitation in nerve. J Physiol.

[52] Swartz KJ (2008). Sensing voltage across lipid membranes. Nature.

[53] Long SB, Tao X, Campbell EB, MacKinnon R (2007). Atomic structure of a voltage-dependent K^+^ channel in a lipid membrane-like environment. Nature.

[54] Long SB, Campbell EB, Mackinnon R (2005). Crystal structure of a mammalian voltage-dependent Shaker family K^+^ channel. Science.

[55] Lu Z, Klem AM, Ramu Y (2001). Ion conduction pore is conserved among potassium channels. Nature.

[56] Alabi AA, Bahamonde MI, Jung HJ, Kim JI, Swartz KJ (2007). Portability of paddle motif function and pharmacology in voltage sensors. Nature.

[57] Xu Y, Ramu Y, Lu Z (2010). A shaker K^+^ channel with a miniature engineered voltage sensor. Cell.

[58] Catterall WA (2010). Signaling complexes of voltage-gated sodium and calcium channels. Neurosci Lett.

[59] Sasaki M, Takagi M, Okamura Y (2006). A voltage sensor-domain protein is a voltage-gated proton channel. Science.

[60] Ramsey IS, Moran MM, Chong JA, Clapham DE (2006). A voltage-gated proton-selective channel lacking the pore domain. Nature.

[61] Murata Y, Iwasaki H, Sasaki M, Inaba K, Okamura Y (2005). Phosphoinositide phosphatase activity coupled to an intrinsic voltage sensor. Nature.

[62] Clayton GM, Altieri S, Heginbotham L, Unger VM, Morais-Cabral JH (2008). Structure of the transmembrane regions of a bacterial cyclic nucleotide-regulated channel. Proc Natl Acad Sci USA.

[63] Chen X, Wang Q, Ni F, Ma J (2010). Structure of the full-length Shaker potassium channel Kv1.2 by normal-mode-based X-ray crystallographic refinement. Proc Natl Acad Sci USA.

[64] Lee SY, Lee A, Chen J, MacKinnon R (2005). Structure of the KvAP voltage-dependent K^+^ channel and its dependence on the lipid membrane. Proc Natl Acad Sci USA.

[65] Lee SY, Banerjee A, MacKinnon R (2009). Two separate interfaces between the voltage sensor and pore are required for the function of voltage-dependent K(+) channels. PLoS Biol.

[66] Aggarwal SK, MacKinnon R (1996). Contribution of the S4 segment to gating charge in the Shaker K^+^ channel. Neuron.

[67] Jiang Y, Ruta V, Chen J, Lee A, MacKinnon R (2003). The principle of gating charge movement in a voltage-dependent K^+^ channel. Nature.

[68] Posson DJ, Selvin PR (2008). Extent of voltage sensor movement during gating of shaker K^+^ channels. Neuron.

[69] Pathak MM, Yarov-Yarovoy V, Agarwal G, Roux B, Barth P, Kohout S, Tombola F, Isacoff EY (2007). Closing in on the resting state of the Shaker K(+) channel. Neuron.

[70] Butterwick JA, MacKinnon R (2010). Solution structure and phospholipid interactions of the isolated voltage-sensor domain from KvAP. J Mol Biol.

[71] Santos JS, Lundby A, Zazueta C, Montal M (2006). Molecular template for a voltage sensor in a novel K^+^ channel. I. Identification and functional characterization of KvLm, a voltage-gated K^+^ channel from Listeria monocytogenes. J Gen Physiol.

[72] Zhao Y, Scheuer T, Catterall WA (2004). Reversed voltage-dependent gating of a bacterial sodium channel with proline substitutions in the S6 transmembrane segment. Proc Natl Acad Sci USA.

[73] Sesti F, Rajan S, Gonzalez-Colaso R, Nikolaeva N, Goldstein SA (2003). Hyperpolarization moves S4 sensors inward to open MVP, a methanococcal voltage-gated potassium channel. Nat Neurosci.

[74] Tombola F, Pathak MM, Isacoff EY (2005). Voltage-sensing arginines in a potassium channel permeate and occlude cation-selective pores. Neuron.

[75] Sokolov S, Scheuer T, Catterall WA (2005). Ion permeation through a voltage- sensitive gating pore in brain sodium channels having voltage sensor mutations. Neuron.

[76] Starace DM, Bezanilla F (2001). Histidine scanning mutagenesis of basic residues of the S4 segment of the shaker K^+^ channel. J Gen Physiol.

[77] Starace DM, Bezanilla F (2004). A proton pore in a potassium channel voltage sensor reveals a focused electric field. Nature.

[78] Catterall WA (2010). Ion channel voltage sensors: structure, function, and pathophysiology. Neuron.

[79] Vieira-Pires RS, Morais-Cabral JH (2010). 3(10) helices in channels and other membrane proteins. J Gen Physiol.

[80] Yarov-Yarovoy V, DeCaen PG, Westenbroek RE, Pan CY, Scheuer T, Baker D, Catterall WA (2012). Structural basis for gating charge movement in the voltage sensor of a sodium channel. Proc Natl Acad Sci USA.

[81] Tao X, Lee A, Limapichat W, Dougherty DA, MacKinnon R (2010). A gating charge transfer center in voltage sensors. Science.

[82] Henrion U, Renhorn J, Borjesson SI, Nelson EM, Schwaiger CS, Bjelkmar P, Wallner B, Lindahl E, Elinder F (2012). Tracking a complete voltage-sensor cycle with metal-ion bridges. Proc Natl Acad Sci USA.

[83] Lundby A, Santos JS, Zazueta C, Montal M (2006). Molecular template for a voltage sensor in a novel K^+^ channel. II. Conservation of a eukaryotic sensor fold in a prokaryotic K^+^ channel. J Gen Physiol.

[84] Zheng H, Liu W, Anderson LY, Jiang QX (2011). Lipid-dependent gating of a voltage-gated potassium channel. Nat Commun.

[85] Gribble FM, Reimann F (2003). Sulphonylurea action revisited: the post-cloning era. Diab tologia.

[86] Kuo A, Gulbis JM, Antcliff JF, Rahman T, Lowe ED, Zimmer J, Cuthbertson J, Ashcroft FM, Ezaki T, Doyle DA (2003). Crystal structure of the potassium channel KirBac1.1 in the closed state. Science.

[87] Nishida M, Cadene M, Chait BT, MacKinnon R (2007). Crystal structure of a Kir3.1-prokaryotic Kir channel chimera. EMBO J.

[88] Tao X, Avalos JL, Chen J, MacKinnon R (2009). Crystal structure of the eukaryotic strong inward-rectifier K^+^ channel Kir2.2 at 3.1 A resolution. Science.

[89] Whorton MR, MacKinnon R (2011). Crystal structure of the mammalian GIRK2 K^+^ channel and gating regulation by G proteins, PIP2, and sodium. Cell.

[90] Bavro VN, De Zorzi R, Schmidt MR, Muniz JR, Zubcevic L, Sansom MS, Venien-Bryan C, Tucker SJ (2012). Structure of a KirBac potassium channel with an open bundle crossing indicates a mechanism of channel gating. Nat Struct Mol Biol.

[91] Whorton MR, MacKinnon R (2013). X-ray structure of the mammalian GIRK2-betagamma G-protein complex. Nature.

[92] Zubcevic L, Bavro VN, Muniz JR, Schmidt MR, Wang S, De Zorzi R, Venien-Bryan C, Sansom MS, Nichols CG, Tucker SJ (2014). Control of KirBac3.1 potassium channel gating at the interface between cytoplasmic domains. J Biol Chem.

[93] Hansen SB, Tao X, MacKinnon R (2011). Structural basis of PIP2 activation of the classical inward rectifier K^+^ channel Kir2.2. Nature.

[94] Raja M (2011). Diverse gating in K^+^ channels: differential role of the pore-helix glutamate in stabilizing the channel pore. Biochem Biophys Res Commun.

[95] Pegan S, Arrabit C, Zhou W, Kwiatkowski W, Collins A, Slesinger PA, Choe S (2005). Cytoplasmic domain structures of Kir2.1 and Kir3.1 show sites for modulating gating and rectification. Nat Neurosci.

[96] Xu Y, Shin HG, Szep S, Lu Z (2009). Physical determinants of strong voltage sensitivity of K(+) channel block. Nat Struct Mol Biol.

[97] Clarke OB, Caputo AT, Hill AP, Vandenberg JI, Smith BJ, Gulbis JM (2010). Domain reorientation and rotation of an intracellular assembly regulate conduction in Kir potassium channels. Cell.

[98] Suh BC, Hille B (2008). PIP2 is a necessary cofactor for ion channel function: how and why?. Annu Rev Biophys.

[99] D’Avanzo N, Cheng WW, Wang S, Enkvetchakul D, Nichols CG (2010). Lipids driving protein structure? Evolutionary adaptations in Kir channels. Channels.

[100] Inanobe A, Nakagawa A, Matsuura T, Kurachi Y (2010). A structural determinant for the control of PIP2 sensitivity in G protein-gated inward rectifier K^+^ channels. J Biol Chem.

[101] Fink M, Lesage F, Duprat F, Heurteaux C, Reyes R, Fosset M, Lazdunski M (1998). A neuronal two P domain K^+^ channel stimulated by arachidonic acid and polyunsaturated fatty acids. EMBO J.

[102] Blondeau N, Widmann C, Lazdunski M, Heurteaux C (2002). Polyunsaturated fatty acids induce ischemic and epileptic tolerance. Neuroscience.

[103] Brohawn SG, Campbell EB, MacKinnon R (2014). Physical mechanism for gating and mechanosensitivity of the human TRAAK K^+^ channel. Nature.

[104] Lolicato M, Riegelhaupt PM, Arrigoni C, Clark KA, Minor DL (2014). Transmembrane helix straightening and buckling underlies activation of mechanosensitive and thermosensitive K(2P) channels. Neuron.

[105] Piechotta PL, Rapedius M, Stansfeld PJ, Bollepalli MK, Ehrlich G, Andres-Enguix I, Fritzenschaft H, Decher N, Sansom MS, Tucker SJ, Baukrowitz T (2011). The pore structure and gating mechanism of K2P channels. EMBO J.

[106] Dong YY, Pike AC, Mackenzie A, McClenaghan C, Aryal P, Dong L, Quigley A, Grieben M, Goubin S, Mukhopadhyay S, Ruda GF, Clausen MV, Cao L, Brennan PE, Burgess-Brown NA, Sansom MS, Tucker SJ, Carpenter EP (2015). K2P channel gating mechanisms revealed by structures of TREK-2 and a complex with Prozac. Science.

[107] Miller AN, Long SB (2012). Crystal structure of the human two-pore domain potassium channel K2P1. Science.

[108] Brohawn SG, del Marmol J, MacKinnon R (2012). Crystal structure of the human K2P TRAAK, a lipid- and mechano-sensitive K^+^ ion channel. Science.

[109] Brohawn SG, Campbell EB, MacKinnon R (2013). Domain-swapped chain connectivity and gated membrane access in a Fab-mediated crystal of the human TRAAK K^+^ channel. Proc Natl Acad Sci USA.

[110] Jiang Y, Lee A, Chen J, Cadene M, Chait BT, MacKinnon R (2002). Crystal structure and mechanism of a calcium-gated potassium channel. Nature.

[111] Pau VP, Smith FJ, Taylor AB, Parfenova LV, Samakai E, Callaghan MM, Abarca-Heidemann K, Hart PJ, Rothberg BS (2011). Structure and function of multiple Ca^2+^-binding sites in a K^+^ channel regulator of K^+^ conductance (RCK) domain. Proc Natl Acad Sci USA.

[112] Ye S, Li Y, Chen L, Jiang Y (2006). Crystal structures of a ligand-free MthK gating ring: insights into the ligand gating mechanism of K^+^ channels. Cell.

[113] Wu Y, Yang Y, Ye S, Jiang Y (2010). Structure of the gating ring from the human high-conductance Ca^2+^-gated K^+^ channel. Nature.

[114] Yuan P, Leonetti MD, Hsiung Y, MacKinnon R (2012). Open structure of the Ca^2+^ gating ring in the high-conductance Ca^2+^-activated K^+^ channel. Nature.

[115] Yuan P, Leonetti MD, Pico AR, Hsiung Y, MacKinnon R (2010). Structure of the human BK channel Ca^2+^-activation apparatus at 3.0 A resolution. Science.

[116] Albright RA, Ibar JL, Kim CU, Gruner SM, Morais-Cabral JH (2006). The RCK domain of the KtrAB K^+^ transporter: multiple conformations of an octameric ring. Cell.

[117] Roosild TP, Miller S, Booth IR, Choe S (2002). A mechanism of regulating transmembrane potassium flux through a ligand-mediated conformational switch. Cell.

[118] Wang L, Sigworth FJ (2009). Structure of the BK potassium channel in a lipid membrane from electron cryomicroscopy. Nature.

[119] Berkefeld H, Sailer CA, Bildl W, Rohde V, Thumfart JO, Eble S, Klugbauer N, Reisinger E, Bischofberger J, Oliver D, Knaus HG, Schulte U, Fakler B (2006). BKCa-Cav channel complexes mediate rapid and localized Ca^2+^-activated K^+^ signaling. Science.

[120] Jiang Y, Pico A, Cadene M, Chait BT, MacKinnon R (2001). Structure of the RCK domain from the E. coli K^+^ channel and demonstration of its presence in the human BK channel. Neuron.

[121] Lundback AK, Muller SA, Engel A, Hebert H (2009). Assembly of Kch, a putative potassium channel from Escherichia coli. J Struct Biol.

[122] Hellmer J, Zeilinger C (2003). MjK1, a K^+^ channel from M. jannaschii, mediates K^+^ uptake and K^+^ sensitivity in E. coli. FEBS Lett.

[123] Ptak CP, Cuello LG, Perozo E (2005). Electrostatic interaction of a K^+^ channel RCK domain with charged membrane surfaces. Biochemistry.

[124] Cao Y, Pan Y, Huang H, Jin X, Levin EJ, Kloss B, Zhou M (2013). Gating of the TrkH ion channel by its associated RCK protein TrkA. Nature.

[125] Roosild TP, Castronovo S, Miller S, Li C, Rasmussen T, Bartlett W, Gunasekera B, Choe S, Booth IR (2009). KTN (RCK) domains regulate K^+^ channels and transporters by controlling the dimer-hinge conformation. Structure.

[126] Roosild TP, Castronovo S, Healy J, Miller S, Pliotas C, Rasmussen T, Bartlett W, Conway SJ, Booth IR (2010). Mechanism of ligand-gated potassium efflux in bacterial pathogens. Proc Natl Acad Sci USA.

[127] Smith FJ, Pau VP, Cingolani G, Rothberg BS (2013). Structural basis of allosteric interactions among Ca^2+^-binding sites in a K^+^ channel RCK domain. Nat Commun.

[128] Kuang Q, Purhonen P, Jegerschold C, Hebert H (2014). The projection structure of Kch, a putative potassium channel in Escherichia coli, by electron crystallography. Biochim Biophys Acta.

[129] Kuang Q, Purhonen P, Jegerschold C, Koeck PJ, Hebert H (2015). Free RCK Arrangement in Kch, a Putative Escherichia coli Potassium Channel, as Suggested by Electron Crystallography. Structure.

[130] Pettersen EF, Goddard TD, Huang CC, Couch GS, Greenblatt DM, Meng EC, Ferrin TE (2004). UCSF Chimera—a visualization system for exploratory research and analysis. J Comput Chem.

[131] Payandeh J, Scheuer T, Zheng N, Catterall WA (2011). The crystal structure of a voltage-gated sodium channel. Nature.

[132] Zhang X, Ren W, DeCaen P, Yan C, Tao X, Tang L, Wang J, Hasegawa K, Kumasaka T, He J, Clapham DE, Yan N (2012). Crystal structure of an orthologue of the NaChBac voltage-gated sodium channel. Nature.

[133] Liao M, Cao E, Julius D, Cheng Y (2013). Structure of the TRPV1 ion channel determined by electron cryo-microscopy. Nature.

[134] Payandeh J, Gamal El-Din TM, Scheuer T, Zheng N, Catterall WA (2012). Crystal structure of a voltage-gated sodium channel in two potentially inactivated states. Nature.

